# Images in cardiovascular disease: a rare case of perforated abscess of mitral valve after Infective Endocarditis (IE)

**DOI:** 10.1186/s44348-024-00006-5

**Published:** 2024-09-05

**Authors:** Agata Niedźwiedzka, Agnieszka Pawlak, Piotr Suwalski, Robert Gil

**Affiliations:** 1 Cardiology Department, National Medical Institute of the Ministry of the Interior and Administration, Wołoska Street 137, Warsaw, 02-507 Poland; 2 Mossakowski Medical Research Centre, Polish Academy of Sciences, Warsaw, Poland

A 45-year-old patient presented to the emergency department reporting an episode of severe dyspnoea accompanied by chest discomfort that occurred at night. In addition, the patient periodically reported a feeling of a fast heartbeat, worsening of exercise tolerance and night sweats for several years. Medical history included an upper respiratory infection 3 weeks ago, uncontrolled hypertension, obesity, alcohol abuse and *Streptococcus pneumoniae* bacteraemia 5 years ago. Physical examination revealed loud holosystolic murmur at the apex. Electrocardiogram showed normal sinus rhythm and Q wave in II, III, aVF. Laboratory tests presented elevated troponin I (116.5 pg/mL) and N-terminal-pro-brain natriuretic peptide (591 pg/mL), inflammatory markers were within normal range. Transesophageal echocardiography revealed spontaneous echo contrast, bicuspid aortic valve, severe eccentric aortic regurgitation, mitral annular dilatation and a 15 × 15 mm capsule-like structure within the anterior leaflet of the mitral valve, possibly corresponding to a drained abscess or aneurysm. The capsule was perforated in several places (through which it was filling with a wave of regurgitation from the aortic valve) and was moving from the left atrium to the left ventricle through the anterior leaflet perforation causing a severe mitral regurgitation (Fig. 1A-C, Movies 1, 2, 3, 4, 5 and 6). The patient was qualified for cardiosurgical treatment where degeneration of aortic valve and capsule on the anterior leaflet of mitral valve were discovered (Fig. 1D). Both valves were replaced with biologic prosthesis. The blood culture test was positive for *Staphylococcus capitis*. Valve tissue culture test came back negative. *S. capitis* is a part of normal skin flora and it is responsible for < 10% of native valve endocarditis [1–3]. Given the lack of signs of current inflammation, based on medical knowledge we suspect the structure is related to the bacteraemia 5 years ago.

**Fig. 1:**
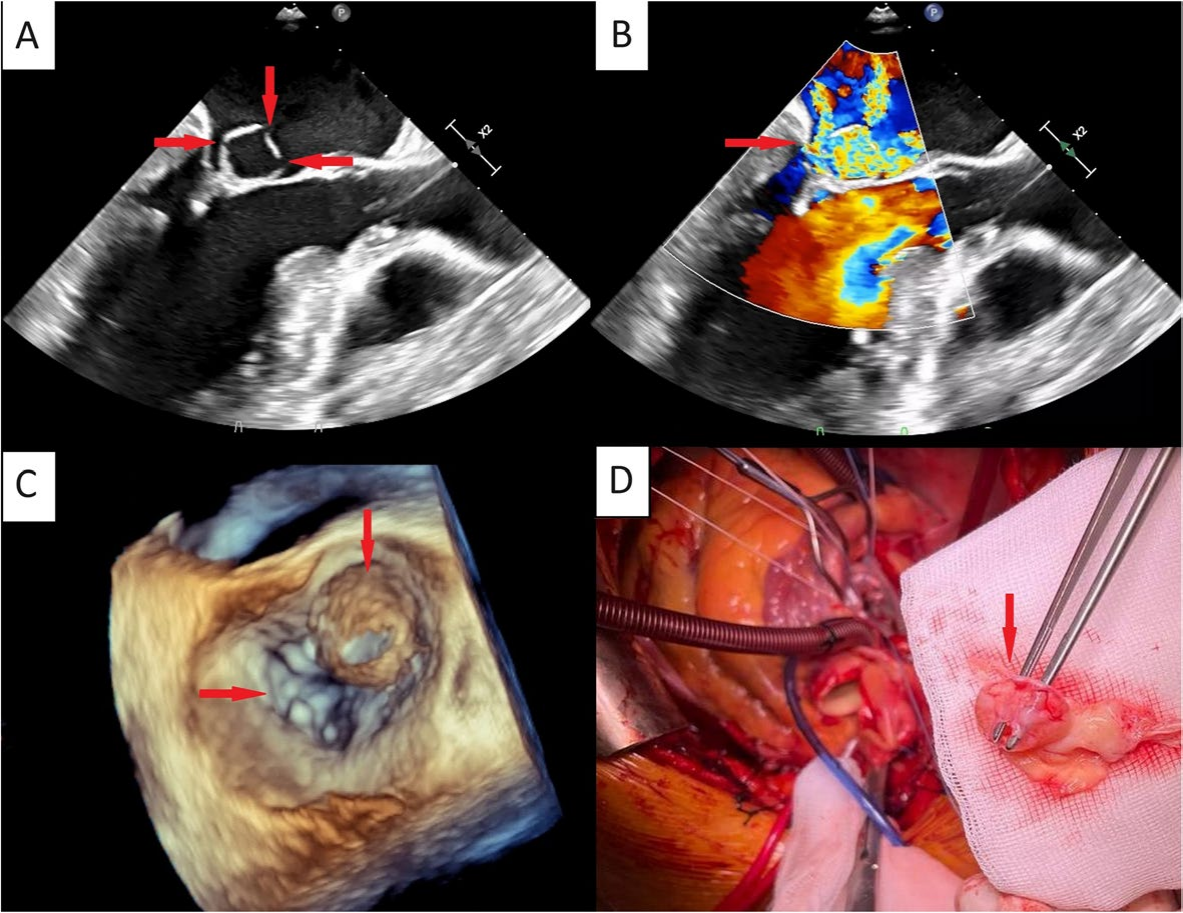
Transesophageal echocardiography revealed: **A** perforated abscess of the anterior mitral valve leaflet; **B** color turbulence due to wave of regurgitation from the aortic valve filling the perforated capsule. Three-dimensional echocardiographic imaging showed: **C** perforated capsule within the mitral valve; **D** intraoperative picture of the capsule

The authors confirm that written consent for submission and publication of this case report including images and associated text has been obtained from the patient in line with Committee on Publication Ethics guidance.

## Supplementary Information

**Additional file 1: Movie 1.** On the top video we can see the movement of the perforated abscess of the anterior mitral valve leaflet—from the left atrium to the left ventricle through the anterior leaflet perforation. The bottom video shows perforated abscess filling with a wave of regurgitation from the aortic valve.

**Additional file 2: Movie 2.** Three-dimensional imaging presenting movement of the perforated abscess and the mitral valve.

**Additional file 3: Movie 3.** Three-dimensional imaging presenting movement of the perforated abscess in the left atrium.

**Additional file 4: Movie 4.** Transthoracic echocardiography- the parasternal shot axis view.

**Additional file 5: Movie 5.** Transthoracic echocardiography- the apical 4-chamber view: left ventricular ejection fraction 65%, left ventricular index 3.1 cm/m2. 

**Additional file 6: Movie 6.** Transthoracic echocardiography- the parasternal long axis view: bicuspid aortic valve (fusion of noncoronary and left coronary cusps), left coronary cusp prolapse, annulus 25 mm, sinotubular junction 27 mm.
